# Correction: Quantum curcumin mediated inhibition of gingipains and mixed-biofilm of *Porphyromonas gingivalis* causing chronic periodontitis

**DOI:** 10.1039/c8ra90104j

**Published:** 2018-12-21

**Authors:** Ashish Kumar Singh, Shivangi Yadav, Kavyanjali Sharma, Zeba Firdaus, Prerana Aditi, Kaushik Neogi, Monika Bansal, Munesh Kumar Gupta, Asheesh Shanker, Rakesh Kumar Singh, Pradyot Prakash

**Affiliations:** Bacterial Biofilm and Drug Resistance Research Laboratory, Department of Microbiology, Institute of Medical Sciences, Banaras Hindu University Varanasi 221005 India pradyot_micro@bhu.ac.in; Molecular Immunology Laboratory, Department of Biochemistry, Institute of Science, Banaras Hindu University Varanasi 221005 India; Department of Pathology, Institute of Medical Sciences, Banaras Hindu University Varanasi 221005 India; Department of Medicinal Chemistry, Institute of Medical Sciences, Banaras Hindu University Varanasi 221005 India; Department of Pharmaceutical Engineering and Technology, Indian Institute of Technology, Banaras Hindu University Varanasi 221005 India; Faculty of Dental Sciences, Institute of Medical Science, Banaras Hindu University Varanasi 221005 India; Department of Bioinformatics, Central University of South Bihar Gaya 824236 Bihar India

## Abstract

Correction for ‘Quantum curcumin mediated inhibition of gingipains and mixed-biofilm of *Porphyromonas gingivalis* causing chronic periodontitis’ by Ashish Kumar Singh *et al.*, *RSC Adv.*, 2018, **8**, 40426–40445.

The authors regret the incorrect naming of the bacterial species *Actinomycetemcomitans viscosus* in the published article. It should be correctly shown as *Actinomyces viscosus* throughout, on pages 40427 (fifth line of last paragraph), 40436 (sixth line of “Individual isolates & mixed” section), 40437 (Table 2) and 40440 (Table 3).

Also, the ATCC number of *A. viscosus* was incorrectly given as 29522 throughout the published article and should be correctly shown as 15987 in the following places: p. 40429 (“Bacterial strains and culture conditions” section and “Minimum inhibitory concentration determination” section), p. 40430 (“Determination of antibiofilm activity using tissue culture plate assay (TCP)” section), p. 40434 (“Antimicrobial assay of quantum curcumin against clinical isolates of *Porphyromonas gingivalis* and select reference strains” section and “Determination of minimum inhibitory concentration” section), p. 40435 (“Growth rate analysis” section), p. 40437 (Table 2), and on p. 40438 (caption to Fig. 7 (twice), caption to Fig. 8, and sixth line of “Discussion” section).

An error was also present in the published article in the co-author name spelling for K. Sharma. The correct spelling of the author names is as shown here.

The Royal Society of Chemistry apologises for these errors and any consequent inconvenience to authors and readers.

## Supplementary Material

